# Image Quality Comparison of Three 3D Mobile X-Ray Imaging Guidance Devices Used in Spine Surgery: A Phantom Study

**DOI:** 10.3390/s24216883

**Published:** 2024-10-26

**Authors:** Djamel Dabli, Cécile Salvat, Isabelle Fitton, Claire Van Ngoc Ty, Peggy Palanchon, Jean-Paul Beregi, Joël Greffier, Lama Hadid-Beurrier

**Affiliations:** 1 Department of Medical Imaging, IMAGINE UR UM 103, Montpellier University, Nimes University Hospital, Bd Prof Robert Debré, CEDEX 9, 30029 Nîmes, France; 2 Medical Physics and Radiation Protection Department, APHP Lariboisière University Hospital, 75010 Paris, Francelama.hadid@aphp.fr (L.H.-B.); 3 Department of Radiology, Georges Pompidou European Hospital, Paris Cité University, APHP, 75015 Paris, France; 4 Department of Radiodiagnostics, CHU Angers, 4 Rue Larrey, 49933 Angers, France

**Keywords:** 3D imaging, spine surgery, noise power spectrum, task transfer function

## Abstract

An image-quality CT phantom was scanned with three different 3D X-ray imaging guidance devices in the operating theatre: O-Arm, Loop-X, and Airo TruCT. Default acquisition and reconstruction parameters for lumbar spine procedures were used on each device. The tube current was set to a dose level of around 27 mGy. A task-based image quality assessment was performed by calculating the noise power spectrum (NPS) and task transfer function (TTF). A detectability index (d’) was calculated for three simulated bone lesions. The noise magnitude of the O-Arm was higher than the Airo TruCT, and the Loop-X had higher noise than the Airo TruCT. The highest average NPS frequency was for the O-Arm images, and the lowest was for the Loop-X. The TTFs at 50% values were similar for the Airo TruCT and Loop-X devices. Compared to Airo TruCT, the TTF at 50% value increased with the O-Arm by 53.12% and 41.20% for the Teflon and Delrin inserts, respectively. Compared to Airo TruCT, the d’ value was lower with Loop-X by −26.73%, −27.02%, and −23.95% for lytic lesions, sclerotic lesions, and high-density bone, respectively. Each 3D-imaging spine surgery guidance device has its own strengths and weaknesses in terms of image quality. Cone-beam CT systems apparently offer the best compromise between noise and spatial resolution for spine surgery.

## 1. Introduction

Three-dimensional (3D) pre- or intra-operative imaging is a fast-growing technique mainly used in neurosurgery and orthopedic/trauma surgery [1,2]. Over the years, improvements in technology have led to the development of 3D image reconstruction algorithms and flat-panel detectors, which have significantly improved image quality. Unlike two-dimensional (2D) projection images, which offer a limited field of view, spatial resolution, and noise, 3D images can better depict challenging anatomical features. The main benefits of intraoperative 3D imaging have been demonstrated in spine surgery to tackle the challenge of joint surface reconstructions [3,4]. Studies have shown that 3D imaging increases the accuracy of pedicle screw or implant placement and reduces the number of surgical revisions and operative time compared to conventional C-arms [5,6]. Similarly, the use of navigation seems to be particularly beneficial for procedures which are difficult to visualize [7,8]. Other applications have also been validated, such as stereotaxic brain surgery [9], treatment of intracranial aneurysms [10], epilepsy [3], or complex joint fractures including the sacroiliac joint, wrist [11], tibia [12], acetabulum [13,14], and syndesmosis [15]. Thus, 3D imaging has become very common in minimally invasive surgery and provides a basis for surgical planning, procedure accuracy, and patient safety.

Many mobile 3D-imaging devices are available in operating rooms. Each system offers a unique technical approach and has its own advantages and pitfalls. These systems can be classified into three categories: C-arm geometry with ~200° rotation, O-ring geometry with 360° rotation, and Computed Tomography (CT) scanners. C-arm and O-ring devices use a cone-shaped beam to generate a 3D CT-like dataset referred to as cone-beam CT (CBCT). The O-arm^®^ (Medtronic, Minneapolis, MN, USA), Airo TruCT^®^ (Stryker, Kalamazoo, MI, USA), and Loop-XTM (Brainlab AG, Munich, Germany) are three mobile systems designed and used for 3D imaging in operating rooms. The O-arm system is based on an X-ray tube coupled with a flat-panel detector, which rotates through 360 degrees to acquire CBCT images. The Airo TruCT system is a 32-sclice CT scanner combining self-propelled mobility with a large inner bore. The Loop-X is a mobile imaging robot equipped with an independent moving imaging source and detector panels to allow for flexible patient positioning, and non-isocentric imaging to focus on the region of interest. It features 2D planar, 3D-CBCT, and fluoroscopic imaging.

Several studies have shown the benefits of the three systems in image-guided surgery to improve patient safety [5,16,17,18,19]. Additionally, a few studies have been concerned about patient and staff exposure to ionizing radiation and subjective image quality [1,19,20,21,22,23,24]. However, to the best of our knowledge, no previous studies have ever assessed the image quality of these three devices objectively with respect to radiation dose using a task-based image quality evaluation.

This work aimed to compare three different mobile 3D intraoperative systems in terms of image quality and possible dose reduction. To complete this study, a task-based image quality assessment was conducted using an image quality phantom for spine applications. We examined image resolution using task transfer function (TTF), noise using the noise power spectrum (NPS), and detectability (d’).

## 2. Materials and Methods

**Figure 1:**
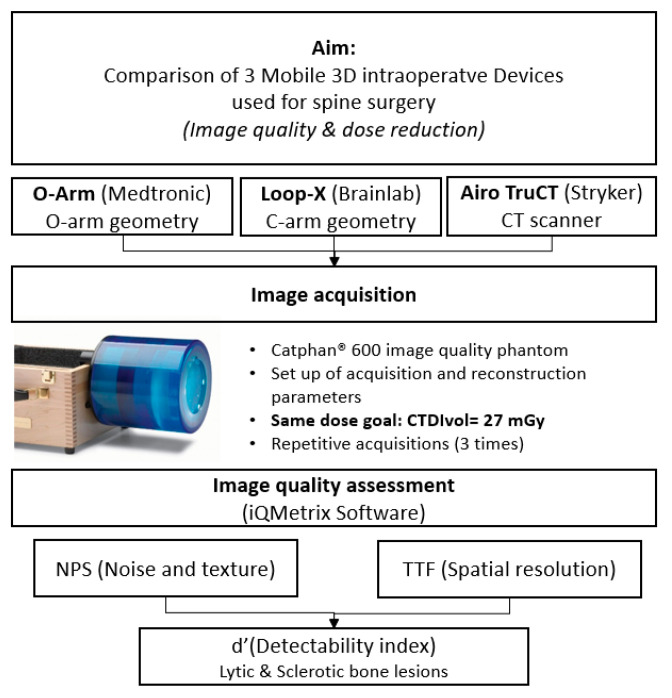
Summary diagram of the study objective and steps of methodology.

Figure 1 shows a summary diagram of the study objective and methodological steps.

### 2.1. Acquisition and Reconstruction Parameters

A Catphan^®^ 600 image quality CT phantom (The Phantom Laboratory, Salem, NY, USA) was scanned with the three 3D X-ray imaging guidance devices used in the operating theatre: the O-Arm (Medtronic, Minneapolis, MN, USA), the Loop-X (Brainlab AG, Germany), and the Airo^®^ TruCT^®^ MobiCT-32 (Stryker, MI, USA). Helical acquisitions were performed with the Airo TruCT scanner with a pitch factor of 1.415. The technical specifications of these devices are given in Table 1. Clinical acquisition and reconstruction parameters for lumbar spine procedures were used for each device (Table 2).

For Airo TruCT, an air calibration was performed before acquisition according to the manufacturers ‘requirements.

Clinical acquisition and reconstruction parameters for lumbar spine procedures were used for each device (Table 2).

The tube current was set to obtain a weighted CT dose index (CTDIw) or volume CT dose index (CTDIvol) of around 27 mGy. This value is the standard national dose for lumbar spine CT scans [25]. Acquisitions were repeated three times on each device to increase the accuracy of the image quality analyses.

### 2.2. Task-Based Image Quality Assessment

A task-based image quality assessment was performed using iQMetrix-CT software (version 1.1, SFPM) [26] to assess noise magnitude and texture using the NPS and spatial resolution using the TTF [27]. Images from the three acquisitions were pooled per equipment to calculate the NPS and TTF. This increased the number of images used to calculate these metrics and reduced the impact of noise on TTF calculations [28]. Finally, the detectability index (d′) was computed to assess the ability of the radiologist to detect three types of lesions encountered in lumbar spine treatment.

The choice of these metrics was based on their ability to characterize image quality in conditions more similar to clinical use. The NPS allows for the assessment of the average noise in the image, as well as its frequency distribution, thus characterizing image texture, which can also have an impact on image quality through smoothing effects. TTF, on the other hand, enables spatial resolution to be assessed for a contrast and dose level closer to those used in clinical practice. Finally, detectability simulates the surgeon’s ability to perform a given clinical task by integrating into the calculation the impact of noise (NPS), spatial resolution (TTF), the characteristics of the clinical task (contrast, lesion shape, and size), and a visual response function that takes account of image viewing conditions. These metrics have been widely used for CT image quality evaluations and to compare CT and CBCT image quality, as well [29,30].

#### 2.2.1. Noise Power Spectrum

**Figure 2:**
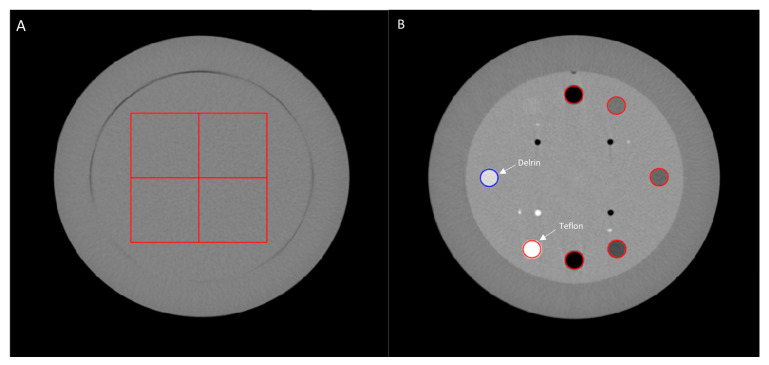
Catphan phantom images: (**A**) a uniform module (CTP 486) with the region of interest for NPS calculation. (**B**) Sensitometry module (CT404) with the region of interest placed on Delrin and Teflon inserts for TTF calculation.

For each device, the NPS was computed on the uniform module (CTP 486) of the Catphan 600 phantom using 30 consecutive slices. Four square regions of interest (ROIs) measuring 92 × 92 pixels with a margin of 30 pixels were placed in this uniform module (Figure 2A), and the NPS was calculated according to the following formula:

(1) $${NPS}_{2D}\left(f_{x},f_{y}\right)=\frac{{∆}_{x}{∆}_{y}}{L_{x}L_{y}}\frac{1}{N_{ROI}}\sum_{i=1}^{N_{ROI}}{\left|{FFT}_{2D}\left\{{ROI}_{i}\left(x,y\right)-{FIT}_{i}(x,y)\right\}\right|}^{2}$$

where Δx and Δy are the pixel sizes in the x- and y-directions; FFT is the Fast Fourier Transform; Lx and Ly are the lengths of the ROIs in the x- and y-directions; NROI is the number of ROIs; ROIi (x,y) is the mean pixel value measured for a ROI at the position (x, y); and FITi (x,y) is a second-order polynomial fit of ROIi (x,y).

The noise magnitude and the average spatial frequency (f_av_) were calculated to quantify the noise level and noise texture, respectively. The following formula was used to compute the f_av_ values:

(2) $$f_{av}=\frac{\int f.NPS(f)df}{\int NPS\left(f\right)df}$$

where f is the radial spatial frequency and NPS(f) is the radially re-binned/average 1D NPS [28].

#### 2.2.2. Task-Based Transfer Function

The TTF was assessed using Delrin and Teflon inserts on the sensitometry module (CT404) of the Catphan phantom (Figure 2B) according to the methodology reported by Richard et al. [27].

A circular ROI was placed around the inserts, and a circular-edge technique was used to measure the Edge Spread Function (ESF) computed using 15 consecutive slices. The ESF was obtained by calculating the radius of each pixel from the centre of each pixel of the insert. The Line Spread Function (LSF) was calculated by derivation from the ESF. Finally, the TTF was computed from the LSF normalized Fourier transformation.

#### 2.2.3. Detectability Index

A detectability index (d’) was calculated using a non-prewhitening observer model with an eye filter (NPWE):

(3) $${d^{\prime }}_{NPWE}^{2}=\frac{\left[\iint \left|W(u,v)\right|2.TTF{\left(u,v\right)}^{2}.E{\left(u,v\right)}^{2}dudv\right]2}{\iint {\left|W\left(u,v\right)\right|}^{2}.TTF{\left(u,v\right)}^{2}.NPS\left(u,v\right).{E(u,v)}^{4}dudv}$$

where u and v are spatial frequencies in the x- and y-directions, E is the eye filter that models the human visual system sensitivity to different spatial frequencies [31], and W(u,v) is the task function defined as:

(4) $$W=\left|F\left\{h_{1}\left(x,y\right)-h_{2}\left(x,y\right)\right\}\right|$$

where F is the Fourier transform, and h_1_ (x,y) and h_2_ (x,y) correspond to the object present and object absent hypotheses, respectively.

The eye filter was modelled according to the visual response function using a zoom factor of 1.5 and a 500 mm viewing distance.

Two task functions measuring 5 mm in diameter were simulated to represent the three types of bone lesion. The task function was assumed to represent a circular signal, with a contrast value of 100 HU for the lytic bone lesion, 350 HU for the sclerotic lesion, and 900 HU for high-density bone. The TTF results of the Delrin insert were used for the lytic and sclerotic lesions, and the TTF results of the Teflon insert were used for the high-density bone. These parameters had already been defined thanks to a previous study [32].

## 3. Results

### 3.1. Noise Power Spectrum

**Figure 3:**
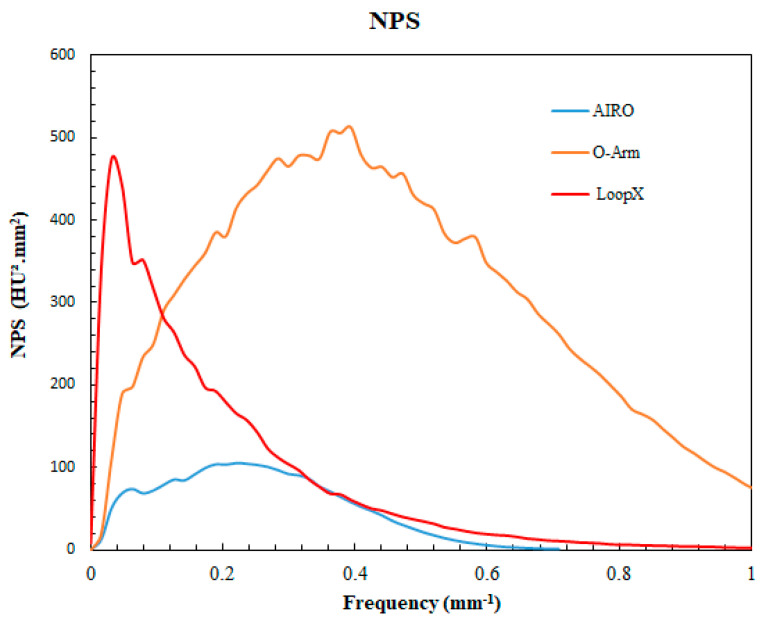
Noise power spectrum results obtained for the three devices (Airo TruCT, Loop-X, and O-Arm).

The NPS curves are presented in Figure 3, and the noise magnitude, f_av_, and f_peak_ results in Table 3.

The highest noise magnitude value was found for the O-Arm, and the lowest for the Airo TruCT. The O-Arm’s noise magnitude was 3.79 times higher than the Airo TruCT, and the Loop-X had 1.62 times higher noise than the Airo TruCT.

Concerning the f_av_ results, the highest value was observed for the O-Arm images and the lowest for the Loop-X. Compared to the Airo TruCT, the f_av_ was 2.13 times higher with the O-Arm and 1.29 times lower with the Loop-X. Similar results were found for f_peak_, with the highest value for the O-Arm and the lowest value for Loop-X.

### 3.2. Task-Based Transfer Function

**Figure 4:**
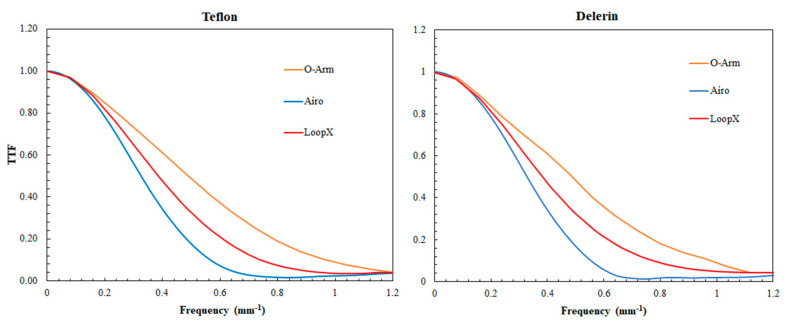
Task transfer function for the three devices (Airo TruCT, Loop-X, and O-Arm) for two inserts: Teflon and Delrin.

The results for TTF are depicted in Figure 4 and Table 4.

For all devices, the TTF_50_ values were similar for both Teflon and Delrin inserts.

Compared to the Airo TruCT, the TTF_50_ value increased with the O-Arm by 53.12% and 41.20% for the Teflon and Delrin inserts, respectively. A smaller increase was found with the Loop-X compared to Airo TruCT, with 21.8% and 9.0% for Teflon and Delrin, respectively. TTF_50_ values were higher with the O-Arm compared to Loop-X for both inserts.

### 3.3. Detectability Index

The results of the detectability index for the three simulated lesions are shown in Table 5 for the three imaging devices.

For all three devices, the highest d’ values were found with high-density bone and the lowest values were observed with lytic lesions.

Compared to the Airo TruCT, the d’ value decreased with Loop-X by −26.73, −27.02, and −23.95% for lytic lesions, sclerotic lesions, and high-density bone. Higher differences were found with the O-Arm, decreasing the d’ by −76.80%, −76.82%, and −76.18%, respectively.

## 4. Discussion

In this phantom study, the image quality of three intraoperative 3D X-ray imaging devices used in the operating theatre was compared. For this purpose, a task-based image quality assessment was made, including calculations of the noise power spectrum (NPS), task transfer function (TTF), and detectability of three simulated bone lesions. The results show that the Airo TruCT provided a lower image noise level and the O-Arm device provided a higher level. However, the O-Arm presented a higher average frequency (f_av_) than the Loop-X, which provided the lowest value for f_av_. Similar spatial resolution values were found for the Airo TruCT and Loop-X, but higher values were observed for the O-Arm. The detectability index values were highest with the Airo TruCT and lowest with the O-Arm for all of the simulated lesions.

The results show that the lowest noise level was obtained with the Airo TruCT. This is mainly explained by the low contribution of scattered radiation to image formation for this scanner, compared to the other two CBCT devices. In fact, the Airo TruCT beam is a 32 × 1 mm wide fan beam, whereas the beam used on CBCT is a wide beam exceeding 20 cm at the isocenter. This wider beam increases the ratio between the quantity of scattered photons and primary photons reaching the detector, which is around 0.2 for a scanner with 40 mm collimation and 3 for a CBCT [33]. As scattered photons contribute to image quality degradation and increased image noise, reducing them helps to reduce noise. In addition, the results show that the Loop-X system provides less noise than the O-Arm, but has a lower average NPS frequency. Low f_av_ reflects a change in noise texture, sometimes producing very smooth and blotchy images [34,35,36,37]. This difference in texture can be linked mainly to image post-processing and denoising methods. These methods differ between the two devices, resulting in moderate denoising and image texture preservation for the O-Arm and significant denoising with an impact on image texture for the Loop-X.

The images with the best spatial resolution were those obtained with the O-Arm, whereas the lowest resolution was observed with the Airo TruCT. These results may be explained by the intrinsic characteristics of these devices. The main factors affecting spatial resolution are focal spot dimensions, detector types, and detector matrix sizes. As far as focal spot size is concerned, both CBCT devices allow for the use of a smaller size than the Airo TruCT device. A small focal spot improves the spatial resolution by limiting the effect of geometric blurring [38]. The differences in spatial resolution observed between the O-Arm and the Loop-X may be explained by the differences in detection technology. Indeed, the O-Arm uses amorphous silicon with a high matrix size (2048 × 1536), whereas the Loop-X uses a 1024 × 1024 matrix, which may explain the better TTF_50_ offered by the O-Arm.

For the detectability index, the highest detectability was observed with the Airo TruCT and the lowest with the O-Arm for the three defined clinical tasks. In fact, the d’ outcome is a consequence of the NPS and TTF results. However, as the NPS has a high impact on calculating the detectability index [39], the device with the lowest noise level was the one with the best detectability.

From a clinical viewpoint, the results obtained in this study show that the three X-ray imaging guidance systems for spine surgery each have their own advantages and drawbacks in terms of image quality. To detect low-contrast lesions such as lytic lesions, the Airo TruCT seems to be the preferred system thanks to its low noise level, although it also has the lowest spatial resolution. For high-contrast lesions such as sclerotic or bone lesions, the Airo TruCT system still offers the best detectability thanks to its low noise level, but, given the high contrast, noise in these cases becomes less of a problem, and spatial resolution becomes the main criterion. In this instance, then, the O-Arm seems to give better results in terms of spatial resolution than the Loop-X, but the latter has a lower noise level. It should also be pointed out that, compared to the Airo TruCT device, CBCT systems are more sensitive to artefacts such as scatter artefacts, cone-beam artefacts, and beam-hardening artefacts [40], particularly in the presence of dense materials. In addition to image quality performance, it is important to consider other parameters related to the operating theatre environment, as stated by Rossi et al. (2022) [41]. The device’s ease of mobility, maneuverability, compatibility with navigation software, and availability of optimization tools are all important criteria to take into account. CBCT systems like the Loop-X have the advantage of greater clearance and easier handling than the other two systems. In terms of tools for optimizing patient dose and image quality, the Airo TruCT scanner is equipped with an intensity modulation system and a metal artefact-correction algorithm, depending on the software version. The CBCT systems compensate for the absence of modulation by offering a wide range of acquisition and reconstruction protocols so that operators can choose the one best suited to the procedure. All three devices are suitable for all types of spine surgery, with or without navigation [42,43,44]. Nevertheless, the results show that performance in terms of noise and spatial resolution differed, which may advantage devices with high spatial resolution, such as the AIRO and LoopX, for bone surgery, such as lumbar spine surgery, given the high contrast of bone tissue and the need for good spatial resolution.

This study has certain limitations. First, a non-anthropomorphic phantom with anatomical structures that differ from those of a real patient was used for the evaluation of image quality. The image quality assessment was carried out on an image quality phantom to characterize the intrinsic performance of each piece of equipment under identical conditions using objective metrics. The generalization of these results and their confirmation in clinical practice requires further evaluations of patient images by physicians through subjective assessments of image quality. This might highlight the constraint of inter-physician and inter-patient variability, which could be resolved by a large sample of patients. Moreover, this study needs to be supplemented by evaluations of other anatomical regions, particularly those involved in brain surgery. Finally, the radiation dose levels used are not representative of the levels used clinically for spinal surgery, particularly with CBCT. However, in the absence of data on clinical dose levels with CBCT, the national DRL values for CT scans were chosen to compare the image quality of the three devices at equivalent dose levels.

## 5. Conclusions

In conclusion, each 3D imaging device for spine surgery guidance has its own strengths and weaknesses in terms of image quality. For an equivalent patient dose, CBCT systems offer the best compromise between noise and spatial resolution for spine surgery.

## Figures and Tables

**Table 1:** Technical specifications of the three intraoperative imaging systems (Airo TruCT, Loop-X, and O-Arm).

	Airo TruCT	Loop-X	O-Arm
Free space (cm)	107	102–121	96.5
Detector type	Solid-State Array CdWO4	CsI(Tl)	a-Si
Matrix size	512 × 512	1024 × 1024	2048 × 1536
FOV (cm)	25.6–51.2	25–60 (2D)	21.2–39.7
		3–25–48 (3D)	
Generator power (kW)	30	14.4	32
Nominal kV	120	120	150
kVp range	80–120	40–120	40–140 *
mA range	5–250	0.2–8 continuous	10–100 *
		5–120 pulsed	

Focus size (mm)	1 × 1	0.3 (small)	0.6 × 0.9 (small)
		0.6 (large)	1.2 × 1.7 (large)
Total filtration (mmAl)	6.8 (at 120 kVp)	4.4 (at 75 kVp)	4.7 (at 75 kVp)
		Bow-Tie filter 0.3–3 mm Cu	

* For pulsed radiography mode.

**Table 2:** Acquisition and reconstruction parameters used with three imaging devices (Airo TruCT, Loop-X, and O-Arm).

		Airo TruCT	Loop-X	O-Arm
Acquisition parameters	kVp	120	120	120
	Temps de rotation (s)	1	50	7.5
	Pitch factor	1.415	-	-
	Tube current (mAs)	257	480	298
	Acquisition field of view (SFOV) cm	380	350	350
	CTDI_vol_ (mGy)/CTDIw	27	26.7	26.2
Reconstruction parameters	Reconstruction algorithm	FBP	FBP	FBP
	Displayed field of view (DFOV) cm	256	250	250
	Slice thickness (mm)	1	1.2	0.833
	Kernel	Soft	Std	Std

**Table 3:** Noise magnitude, average, and peak frequency results obtained for the three devices (Airo TruCT, Loop-X, and O-Arm).

	Noise Magnitude (HU)	f_av_ (mm^−1^)	f_peak_ (mm^−1^)
Airo TruCT	7.6	0.22	0.26
Loop-X	12.31	0.17	0.03
O-Arm	28.8	0.47	0.36

**Table 4:** Task transfer function at 50% (TTF_50_) for the three devices (Airo TruCT, Loop-X, and O-Arm).

	TTF_50_	
	Teflon	Delrin
Airo TruCT	0.32	0.34
Loop-X	0.39	0.37
O-Arm	0.49	0.48

**Table 5:** Detectability index values of the three simulated lesions with for the three imaging devices (Airo TruCT, Loop-X, and O-Arm).

	d’		
	Lytic	Sclerotic	Bone
Airo TruCT	7.37	25.80	64.22
Loop-X	5.40	18.83	48.84
O-Arm	1.71	5.98	15.30

## Data Availability

Data are available on request from the corresponding author.
